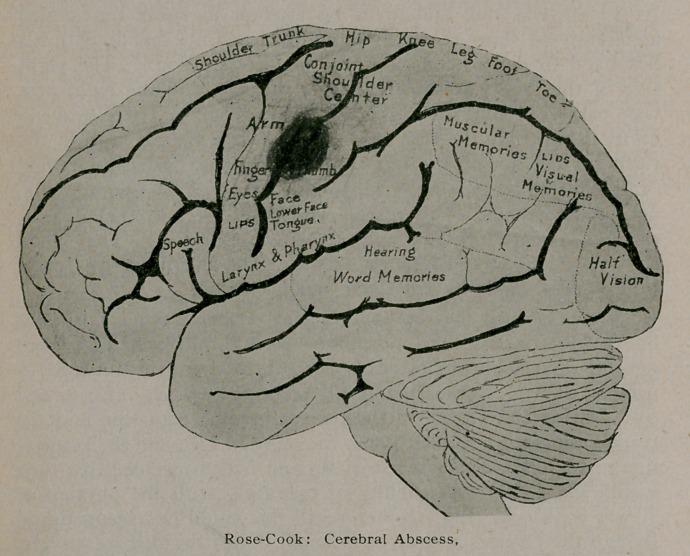# Report of Case of Cerebral Abscess: Operation—Death1Read at the thirty-ninth annual meeting of the Medical Association of Central New York, at Syracuse, October 16, 1906.

**Published:** 1907-02

**Authors:** L. W. Rose, Robert G. Cook

**Affiliations:** Rochester, N. Y.; Rochester, N. Y.


### Report of Case of Cerebral Abscess: Operation—Death.[1]

By L. W. ROSE, M. D., and ROBERT G. COOK, M. D., Rochester, N. Y.

THIS case is of interest not only because of the rarity of cerebral abscess, but also because of the rapidity of the course of the disease and the definite focal symptoms present. The patient, a man aged thirty-seven, single, an insurance agent, was first seen by the writers August 15, 1906. The history, as obtained from the patient and from his friends, shows no evidence of epilepsy or general paralysis and no recent ill health up to a few days previous. There was no history of syphilis nor of injury to the head, nor of supperative disease of the nose or throat. On August 8th he complained of distress in the abdomen, vomited and fell twice when walking. On the 11th he had pain on the right side of the neck and in the right shoulder. On the 14th, the day previous to our first interview with him, some of his friends noticed that "he seemed different," and he had pain in the left side of the head and face.

In the evening he went to a dentist, who extracted an old root of a tooth after an injection of cocaine. The dentist noticed that he gave his card to him instead of telling him his name. The next morning, the 15th, he did not go to the office and his

1. Read at the thirty-ninth annual meeting of the Medical Association of Central New York, at Syracuse, October 16, 1906.

employer found at noon that he was confused and unable to talk properly. When seen by Dr. Rose at five in the evening he seemed slow of speech, stupid, and it was first thought that he was under the influence of some narcotic, though no definite symptoms of drug poisoning could be detected. He was unable to tell his name, he could not tell where his mother lived, but got out of bed and went to his clothes and from a memorandum read where his mother lived. His temperature was normal, his pulse was 60. His friends were told to take him out for a walk and bring him to my office at 8 o'clock that evening.

When he came to the office his symptoms had increased and he presented a more serious clinical picture. His temperature was 99.2 and Dr. Cook was asked to see him. When examined by us he could not tell his own name, the name of the company for which he worked, nor many other names, but he recognized them if spoken and could then repeat them. He could also name correctly common objects if shown to him. He could write only the first two letters of his first name and could not copy writing or printing. He could not tell the day of the week nor the day of the month nor the year. He could read aloud. He showed some confusion and was somewhat irrational about the names of succeeding days, which he could not give. He had two matches and lighted one and then tried to light a cigar with the unlighted one. He realised his aphasia and joked about not being able to remember the name of the insurance company for which he worked.

The pulse was 80, the temperature was 99.2. His tongue was coated and tremulous but was protruded straight. The pupils were equal in size and reacted to light. There was no facial paralysis nor paralysis of the arms or legs. The knee and achilles jerks were normal and there was no Kernig sign. There was a lessening of the sense of pain in both hands and the lower third of both fore arms. He was advised to go to a hospital immediately and reluctantly consented, and a room was engaged for him, but after leaving the office he absolutely refused to go to the hospital and his employer was obliged to allow him to return to his boarding house.

He was next seen by Dr. Cook about 5 o'clock the next day, August 16. The landlady reported that he had not been able to speak for several hours nor to move his right arm, and that he had passed no urine since morning and had seemed to have had pain in the head. He appeared very dull and did not speak at all. There seemed to be complete word deafness and he made no effort to protrude his tongue even when shown what to do. There was partial paralysis and loss of sense of pain in the right arm, and the right side of the face was slightly smoother than the left. The pupils were equal and reacted to light. The pulse was 60 and the axillary temperature was 99.6. He was removed to the Rochester City Hospital in an ambulance and his mother and brother, who were in Kansas City, were telegraphed to come to Rochester.

He was admitted to the hospital at 8:45 P. M., when his pulse was 62, the rectal temperature was 101.6 and the respiration 24. He was restless the greater part of the night and seemed to have severe pain in the head. At 7:30 A. M., the 17th, P 54, T 101.6, R 22; he was given sulphate of magnesia 2 drams, saturated solution of iodide of potash 10 drops, three times a day. At 9:30 A. M., he had a general convulsion. At 12, he was dull and made no attempt to speak but smiled apparently to show recognition, and grasped our hands with his left hand to denote consciousness and recognition. The right side of the face was smoother than the left, and the right arm was paralyzed but the right leg was moved. There was no effort to protrude the tongue. There was a Kernig sign in the right leg but not on the left and there was no Babiniski reflex.

The urine was amber, acid, specific gravity 1030; no albumin, no sugar nor bile. Microscopically there were epithelial cells, calcium oxalate crystals and amorphous urates. The blood showed a leukocyte count of 14,760. The diagnosis was cerebral abscess, made by Dr. Cook and Dr. Rose.

It was learned that his mother was on her way to Rochester but could not arrive until morning. At 1 P. M., he had a second general convulsion followed by others at frequent intervals—namely, at 2:15, 3:15, 3:50, 4:10, 4:20, 6:25, 6:45, 7:15, 8, and 9. During the afternoon the patient became comatose and the rectal temperature rose to 102.6, the pulse to 150, and the respiration to 48. His business associates and friends decided that they would share the responsibility of operating with the physicians. It was 8 o'clock, Aug. 18, before all this could be arranged and though the chances seemed very small it was decided to operate, as he evidently could not live if the convulsions continued. At 9 o'clock, just before the anesthetic was given he had a convulsion which began in the right side of the face and extended to the right arm only.

### OPERATION.

Dr. Cook indicated on the shaven scalp, by the use of tincture iodine, the course of the left Rolandic fissure, and by a drill mark on the skull, the probable centre of the abscess.

The operation by Dr. Rose was a horse-shoe flap of skin and muscle to the periosteum; four openings were made through the skull by a trephine; the skull between these was divided by a Gigli saw; the trephine openings were at the angles of a quadrilateral, three inches apart and equally distant from the central mark previously made on skull. The periosteum of basal line was left intact. When this flap of bone was turned back, there was bulging of the cerebral substance.

The dura was divided by a conical incision. In the centre of the opening there was a softened area, below the surface of the cortex which when punctured by grooved director, gave evidence of pus. This opening was enlarged and pus evacuated leaving a cavity that would admit the end of a man's thumb. This cavity was gently wiped out and drained through the posterior inferior trephine opening, and the flap of bone and skin replaced, and secured by suture line through scalp.

Rose-Cook: Cerebral Abscess.

The abscess was not circumscribed and the encephalitis involved a much larger area than was broken down. The meningites was recent and limited to a small area over centre of abscess.

This probably accounts for the delayed and unilateral Kernig sign.

A very little ether was used to control any continuation of convulsions.

The operation did not restore consciousness and death followed in about one hour after termination of dressing.

The features of this case which seem noteworthy are, first, the unknown origin of the infection. It seemed and still seems probable that it was due to a septic embolus from the tooth or from his decayed teeth. The dentist stated, however, that there was no pus about the tooth extracted. There was no evidence of pus infection in the nose or ear. Second, the rapid course of the disease and the extension of the focal symptoms. Third, the help to an early diagnosis afforded by the blood count in determining pus to be present and the probable cause of the focal symptoms.

## Figures and Tables

**Figure f1:**